# Toward G protein-coupled receptor structure-based drug design using X-ray lasers

**DOI:** 10.1107/S2052252519013137

**Published:** 2019-10-24

**Authors:** Andrii Ishchenko, Benjamin Stauch, Gye Won Han, Alexander Batyuk, Anna Shiriaeva, Chufeng Li, Nadia Zatsepin, Uwe Weierstall, Wei Liu, Eriko Nango, Takanori Nakane, Rie Tanaka, Kensuke Tono, Yasumasa Joti, So Iwata, Isabel Moraes, Cornelius Gati, Vadim Cherezov

**Affiliations:** a Bridge Institute, Departments of Chemistry and Biological Sciences, University of Southern California, Los Angeles, CA 90089, USA; b Linac Coherent Light Source, SLAC National Accelerator Laboratory, Menlo Park, CA 94025, USA; c Department of Physics, Arizona State University, Tempe, AZ 85287, USA; d Department of Chemistry and Physics, La Trobe Institute for Molecular Science, La Trobe University, Melbourne, Victoria 3086, Australia; e School of Molecular Sciences and Biodesign Center for Applied Structural Discovery, Biodesign Institute, Arizona State University, Tempe, AZ 85287, USA; f RIKEN SPring-8 Center, 1-1-1 Kouto, Sayo-cho, Sayo-gun, Hyogo 679-5148, Japan; g Department of Cell Biology, Graduate School of Medicine, Kyoto University, Yoshidakonoe-cho, Sakyo-ku, Kyoto 606-8501, Japan; h Department of Biological Sciences, Graduate School of Science, The University of Tokyo, 2-11-16 Yayoi, Bunkyo, Tokyo 113-0032, Japan; i Japan Synchrotron Radiation Research Institute, 1-1-1 Kouto, Sayo-cho, Sayo-gun, Hyogo 679-5198, Japan; j National Physical Laboratory, Hampton Road, Teddington TW11 0LW, England; k Research Complex at Harwell, Rutherford Appleton Laboratory, Harwell Science and Innovation Campus, Didcot OX11 0FA, England; l Department of Structural Biology, Stanford University, Stanford, CA 94305, USA; m Biosciences Division, SLAC National Accelerator Laboratory, Menlo Park, CA 94025, USA

**Keywords:** drug discovery, G protein-coupled receptors, serial femtosecond crystallography, X-ray free-electron lasers, structure determination, membrane proteins, protein structure, molecular recognition

## Abstract

Rational structure-based drug design (SBDD) relies on the availability of a large number of co-crystal structures to map the ligand-binding pocket of the target protein and use this information for lead-compound optimization via an iterative process. While SBDD has proven successful for many drug-discovery projects, its application to G protein-coupled receptors (GPCRs) has been limited owing to extreme difficulties with their crystallization. Here, a method is presented for the rapid determination of multiple co-crystal structures for a target GPCR in complex with various ligands, taking advantage of the serial femtosecond crystallography approach, which obviates the need for large crystals and requires only submilligram quantities of purified protein. The method was applied to the human β_2_-adrenergic receptor, resulting in eight room-temperature co-crystal structures with six different ligands, including previously unreported structures with carvedilol and propranolol. The generality of the proposed method was tested with three other receptors. This approach has the potential to enable SBDD for GPCRs and other difficult-to-crystallize membrane proteins.

## Abstract 2

A method is presented for efficient co-crystal structure determination for G protein-coupled receptors taking advantage of serial femtosecond crystallography.

## 1. Introduction   

Structure-based drug design (SBDD) is a powerful approach that can substantially accelerate the process of drug discovery and optimization as well as guide medicinal chemists towards the selection of the most promising lead candidates for subsequent clinical trials (Jazayeri *et al.*, 2015 ▸). While even a single structure of the target protein can be very helpful for the discovery of new compounds, the most successful SBDD programs exploit a large number of co-crystal structures to comprehensively map the ligand-binding pocket and ligand-binding modes in an iterative manner. For example, a recent study on inhibitors of the aspartic protease endothiapepsin has shown that even minor chemical modifications in the ligand can cause dramatic and unexpected changes in the ligand-binding mode (Kuhnert *et al.*, 2015 ▸).

G protein-coupled receptors (GPCRs) are ubiquitous cellular gatekeepers in eukaryotic organisms. They mediate sensory stimuli and cell signaling, regulating all major physiological processes, and therefore have historically been primary targets in the pharmaceutical industry (Allen & Roth, 2011 ▸). GPCRs represent the largest protein superfamily in humans, comprising over 800 members (Lagerström & Schiöth, 2008 ▸) that are commonly grouped into five classes according to sequence similarity. The largest of the classes, class A, accounts for nearly 85% of the GPCR superfamily and includes, among others, aminergic receptors targeted by about a quarter of current prescription drugs (Rask-Andersen *et al.*, 2011 ▸).

Since their discovery several decades ago, GPCRs have become some of the most valuable targets for structural studies. Their high-resolution structure determination has been enabled by a number of breakthroughs in several fields, such as protein engineering (Rosenbaum *et al.*, 2007 ▸; Chun *et al.*, 2012 ▸; Serrano-Vega *et al.*, 2008 ▸), crystallization in the native-membrane-mimicking environment of the lipidic cubic phase (LCP; Landau & Rosenbusch, 1996 ▸; Cherezov, 2011 ▸) and microcrystallography (Cherezov *et al.*, 2009 ▸; Smith *et al.*, 2012 ▸). Combined efforts from several laboratories during the last decade have produced over 300 structures of ∼60 unique GPCRs captured in different signaling states (Pándy-Szekeres *et al.*, 2018 ▸). These structures shed light on ligand-recognition and signal-transduction mechanisms. Despite this impressive progress in structural studies of GPCRs, their crystallization remains extremely challenging and constitutes one of the largest hurdles for SBDD applications.

Most commonly in SBDD, multiple co-crystal structures are obtained by soaking various ligands into crystals of unliganded (apo) proteins. Unfortunately, owing to their highly dynamic nature, a routine approach to the crystallization of apo GPCRs has not yet been established, and the use of ligands remains critical for successful receptor solubilization, purification and crystallization. Even after the structure of the target receptor in complex with one of the ligands has been solved, co-crystal structure determination with other ligands still represents a major challenge, as it often requires an extensive optimization of the receptor purification and crystallization conditions in order to obtain sufficiently large crystals for crystallographic data collection at synchrotron sources. Fully optimized GPCR crystals grown in LCP often tend to be well ordered, but owing to their small size and the rapid onset of radiation damage the acquisition of a complete data set requires screening hundreds of crystals and combining the data from dozens of them. Each of these crystals has to be manually harvested, cryocooled and aligned with the X-ray beam using rastering approaches, making the entire process exceedingly tedious and resource-consuming (Cherezov *et al.*, 2009 ▸).

Most of these problems, however, can be essentially overcome by using the serial femtosecond crystallography (SFX) data-collection approach (Chapman *et al.*, 2011 ▸; Liu *et al.*, 2013 ▸; Mishin *et al.*, 2019 ▸). SFX utilizes extremely bright and short pulses (tens of femtoseconds) produced by an X-ray free-electron laser (XFEL) to enable room-temperature data collection from micrometre-sized crystals, outrunning structure-altering radiation damage in a ‘diffraction-before-destruction’ manner (Neutze *et al.*, 2000 ▸). The requirement of small crystal size in SFX experiments simplifies the crystallization optimization process. Furthermore, SFX offers additional advantages, such as a typically higher resolution, owing to higher order and a lower occurrence of defects in small crystals, in comparison to conventional synchrotron data collection, as well as room-temperature data collection that can potentially reveal important features related to protein dynamics (Fraser *et al.*, 2009 ▸, 2011 ▸). Recent progress in SFX data-processing software has considerably lowered the amount of data that is required for structure determination (Kabsch, 2014 ▸; Uervirojnang­koorn *et al.*, 2015 ▸; White *et al.*, 2016 ▸).

Here, we present a simple and efficient method, dubbed Complex-LCP (**C**rystallization **o**f **m**embrane **p**roteins using transient **l**igand **ex**change in **LCP**), for structure determination of a target GPCR in complex with a panel of different ligands by taking advantage of the SFX method. This represents a major step towards high-throughput GPCR–ligand co-crystal structure determination. We validated our approach using the human β_2_-adrenergic receptor (β_2_AR) that has been extensively studied over the last few decades and structures of which are available in both active and inactive states (Cherezov *et al.*, 2007 ▸; Rasmussen *et al.*, 2011 ▸; Wacker *et al.*, 2010 ▸; Ring *et al.*, 2013 ▸). Our method requires at least three components: a transient ligand, a protein and a ligand of interest. Using timolol or alprenolol as a transient ligand for receptor solubilization and purification, we were able to successfully exchange these ligands during crystallization for six β_2_AR ligands with diverse modes of action (MoAs) ranging from inverse agonism and antagonism to arrestin-biased agonism. Overall, eight β_2_AR structures with resolutions of between 2.4 and 3.4 Å were determined in this work. The exchanged ligands were unambiguously identified in the difference electron-density maps obtained with SFX data. The structures of two of these ligands, carvedilol and propranolol, have not previously been reported in complex with β_2_AR. The general applicability of the method was then demonstrated with adenosine A_2A_ (A_2A_AR), serotonin 1B (5-HT_1B_) and 2B (5-HT_2B_) and MT_1_ melatonin receptors.

## 2. Materials and methods   

### 2.1. Receptor constructs   

The gene sequences of the human receptors β_2_AR, A_2A_AR, 5-HT_1B_, 5-HT_2B_ and MT_1_ were modified to increase protein expression and stability as described previously (Hanson *et al.*, 2008 ▸; Liu *et al.*, 2012 ▸; Wacker *et al.*, 2013 ▸; Wang *et al.*, 2013 ▸; Stauch *et al.*, 2019 ▸). Briefly, the gene for human β_2_AR (UniProt ID P07550) was altered as follows: (i) ICL3 residues 231–262 were replaced with cysteine-less T4 lysozyme to improve protein stability and increase the polar surface area for crystallization, (ii) the C-terminus was truncated at residue 348 and (iii) a point mutation E122^3.41^W was introduced to improve the protein yield and stability (Roth *et al.*, 2008 ▸).

The A_2A_AR (UniProt ID P29274) construct was prepared by replacing ICL3 residues Lys209–Gly218 with a thermostabilized apocytochrome *b*_562_ from *Escherichia coli* (BRIL) and truncating the C-terminal residues 317–412. The 5-HT_2B_ (UniProt ID P41595) construct was generated by replacing ICL3 residues Tyr249–Val313 with BRIL and truncating the N-terminal residues 1–35 and the C-terminal residues 406–481 from the original sequence. Similarly to β_2_AR, a thermostabilizing M144^3.41^W mutation (Roth *et al.*, 2008 ▸) was introduced into 5-HT_2B_. The wild-type human 5-HT_1B_ (UniProt ID P28222) was fused to BRIL by replacing ICL3 residues Leu240–Lys303. The N-terminus of 5-HT_1B_ was truncated at Asn32 to remove all glycosylation sites. In addition, a single point mutation L138^3.41^W (Roth *et al.*, 2008 ▸) was introduced to increase the thermostability.

The MT_1_ (UniProt ID P48039) crystallization construct was obtained by the truncation of 11 N-terminal and 25 C-terminal residues, the replacement of intracellular receptor amino-acid residues 219–227 with the catalytic domain of *Pyrococcus abyssi* glycogen synthase (PGS; UniProt ID Q9V2J8) and the introduction of nine essential, stabilizing point mutations (D73^2.50^N, L95^ECL1^F, G104^3.29^A, F116^3.41^W, N124^3.49^D, C127^3.52^L, W251^6.48^F, A292^7.50^P and N299^8.47^D) (Stauch *et al.*, 2019 ▸).

### 2.2. Expression and purification   

All GPCR constructs were cloned into a modified pFastBac1 expression vector bearing N-terminal HA- and FLAG-tags and expressed using the baculovirus expression system. In brief, the recombinant baculovirus was obtained according to the standard protocols in the Bac-to-Bac system (Invitrogen) and used to infect *Spodoptera frugiperda* (Sf9) insect cells at a multiplicity of infection of 5. The cells were harvested after incubation for 48 h. Insect-cell membranes were disrupted by osmotic shock in a hypotonic buffer consisting of 10 m*M* HEPES pH 7.5, 20 m*M* KCl, 10 m*M* MgCl_2_ and EDTA-free protease-inhibitor cocktail (Roche). The hypotonic wash was repeated once and was followed by a high-salt wash in buffer consisting of 1.0 *M* NaCl, 10 m*M* HEPES pH 7.5, 10 m*M* MgCl_2_, 20 m*M* KCl and EDTA-free protease-inhibitor cocktail (Roche). The high-salt wash was repeated 1–2 times. Extensive washing of the membranes was performed by repeated centrifugation and Dounce homogenization to strip the membranes of soluble and membrane-associated proteins. The nembranes were flash-frozen and stored at −80°C until further use. Prior to solubilization, the prepared membranes were thawed on ice in the presence of 20 µ*M* of the corresponding transient ligand (timolol or alprenolol), 2 mg ml^−1^ iodoacetamide and protease inhibitors. The membranes were then solubilized by incubation in the presence of 0.5%(*w*/*v*) *n*-dodecyl-β-d-maltopyranoside (DDM; Avanti Polar Lipids) and 0.1% cholesteryl hemisuccinate (CHS; Sigma) for 3 h at 4°C. After solubilization, the solution was clarified at 100 000*g* and the resulting supernatant was incubated with TALON IMAC resin overnight at 4°C. The resin was washed with ten column volumes (CV) of wash buffer I (50 m*M* HEPES pH 7.5, 300 m*M* NaCl, 20 m*M* imidazole, 0.1/0.02% DDM/CHS) and 5 CV of wash buffer II (50 m*M* HEPES pH 7.5, 300 m*M* NaCl, 40 m*M* imidazole, 0.05/0.01% DDM/CHS) to remove impurities, followed by elution of the receptor with 25 m*M* HEPES pH 7.5, 300 m*M* NaCl, 200 m*M* imidazole, 10% glycerol, 0.015/0.003% DDM/CHS. The transient ligand (timolol or alprenolol) was maintained at a concentration of 50 µ*M* throughout solubilization, washing and elution. MT_1_–agomelatine was purified as previously described (Stauch *et al.*, 2019 ▸).

### 2.3. Lipidic cubic phase crystallization   

Prior to crystallization, all proteins were concentrated to 20–30 mg ml^−1^. LCP was made by mixing two parts (by volume) of protein solution with three parts of molten lipid (monoolein supplemented with 10% cholesterol by weight). Initial LCP crystallization screening of β_2_AR was performed using an NT8-LCP robot (Formulatrix) in 96-well glass sandwich plates (Marienfield) by dispensing 40 nl LCP drops and covering them with 800 nl precipitant solution. In this way, hit conditions were identified that yielded high concentrations of small crystals (∼5 µm) suitable for SFX data collection. The standard 48-salt screens (Xu, Liu *et al.*, 2011 ▸) supplemented with 2 m*M* of the target ligand (from a 100 m*M* stock in DMSO) in each well were used for the setup in sandwich plates. Control Plate A supplemented with a matching concentration of DMSO (no ligand) in the screen was set up in parallel to identify conditions in which the concentration of the transient ligand is not high enough to generate crystals without introduction of the secondary ligand, and Control Plate B was set up supplemented with 2 m*M* of the transient ligand as a positive control of protein quality and crystallo­genesis.

Crystallizations of 5-HT_1B_ and 5-HT_2B_ in plates were performed similarly to that of β_2_AR. In the case of 5-HT_1B_, washing buffers were supplemented with 20 µ*M* ergotamine (ERG) and the crystallization buffer contained 2 m*M* of one of the exchange ligands (methylergometrine, oxymetazoline, sumatripan or RU-24969). In the case of 5-HT_2B_ the washing buffers were supplemented with 50 µ*M* serotonin, and the precipitant (400 m*M* ammonium chloride, 30% PEG 400, 100 m*M* Tris pH 8) contained one of the exchange ligands (ERG or dihydroergotamine) at 2 m*M*.

Crystals for XFEL data collection were obtained in Hamilton gas-tight syringes using the previously reported procedure (Liu *et al.*, 2014 ▸). Purified β_2_AR in complex with an intermediate ligand (timolol or alprenolol) at a concentration of 25 mg ml^−1^ was reconstituted in LCP as described above. Approximately 5 µl of protein-laden LCP was carefully injected as a continuous filament of ∼400 µm in diameter into a 100 µl syringe filled with 60 µl precipitant solution [0.1 *M* HEPES pH 7.0, 0.1 *M* ammonium sulfate, 30%(*v*/*v*) PEG 400, 2 m*M* ligand] and incubated for 24 h at 20°C.

For the ligand-exchange experiments with A_2A_AR, the protein was purified following the previously published protocols (Liu *et al.*, 2012 ▸) using 50 µ*M* LUF5834 as a transient ligand. Microcrystals in syringes were obtained using 50 m*M* sodium thiocyanate, 100 m*M* sodium citrate pH 4.8, 28%(*v*/*v*) PEG 400 supplemented with 2 m*M* ZM241385. Showers of small (∼5 µm) crystals appeared overnight and were used for data collection at the Linac Coherent Light Source (LCLS).

MT_1_ was crystallized as described previously (Stauch *et al.*, 2019 ▸) but using the ligand agomelatine during purification and a precipitant solution consisting of 60–100 m*M* potassium phosphate monobasic, 100 m*M* HEPES pH 7.0, 32–35%(*v*/*v*) PEG 400, the target ligand 2-phenylmelatonin (2-PMT) at 1 m*M*, 2.5%(*v*/*v*) DMSO, 1.5%(*v*/*v*) propan-2-ol.

After crystals had formed, excess precipitant solution was carefully removed, followed by the addition of ∼3 µl 7.9 MAG (Misquitta *et al.*, 2004 ▸) to absorb the residual precipitant solution. The microcrystal samples were characterized on-site at LCLS using a zoom stereomicroscope (Leica) equipped with linear rotating polarizers.

### 2.4. XFEL data collection   

LCP-SFX data collection for β_2_AR with carazolol, timolol, alprenolol and ICI-118,551, for A_2A_AR with ZM241385 and for MT_1_ with 2-PMT was performed using the CXI instrument (Boutet & Williams, 2010 ▸) at LCLS at SLAC National Accelerator Laboratory, Menlo Park, California, USA. LCLS operated at a wavelength of 1.33 Å (9.5 keV), delivering individual X-ray pulses of 42 fs duration with 10^12^ photons per pulse focused into a spot size of approximately 1.5 µm in diameter using a pair of Kirkpatrick–Baez mirrors. Protein microcrystals in LCP medium were injected into the focus region using the LCP injector (Weierstall *et al.*, 2014 ▸) with a 50 µm diameter nozzle at a flow rate of 0.2 µl min^−1^. Microcrystals ranged in size from 1 to ∼10 µm, with an average size of 5 × 2 × 2 µm. Single-shot diffraction patterns of randomly oriented crystals were recorded at a rate of 7200 patterns per minute (120 Hz) with the 2.3 megapixel Cornell–SLAC Pixel Array Detector (CSPAD; Hart *et al.*, 2012 ▸). The beam was attenuated to ∼10% (9 × 10^10^ photons per pulse) of the full intensity to avoid detector saturation.

SFX data for β_2_AR in complex with propranolol and carvedilol were collected on the BL3 beamline at the SPring-8 Angstrom Compact free-electron LAser (SACLA) in Japan using a multiport charge-coupled device (MPCCD) detector (Tono *et al.*, 2015 ▸). The instrument operated at a wavelength of 1.76 Å (7 keV) with a pulse duration of <10 fs and a repetition rate of 30 Hz. The XFEL pulse (471 µJ per pulse) was focused into a spot size of approximately 1.5 µm in diameter. Data collection at SACLA was guided by a real-time data-processing pipeline (Nakane *et al.*, 2016 ▸) based on *Cheetah* (Barty *et al.*, 2014 ▸) and *CrystFEL* (White *et al.*, 2016 ▸).

The overall time of data collection from eight β_2_AR samples (six ligands and two controls) with a total volume of ∼180 µl was about ∼14.8 h and yielded ∼230 000 indexed patterns (Table 1 ▸). Potential single-crystal diffraction patterns were identified using *Cheetah* with a threshold of 15 potential Bragg peaks (Barty *et al.*, 2014 ▸). Indexing, integration and merging of the crystal diffraction data was performed using *CrystFEL* (White *et al.*, 2016 ▸), which involved application of the indexing algorithms in *MOSFLM* (Leslie, 2006 ▸), *XDS* (Kabsch, 2010 ▸) and *DIRAX* (Duisenberg, 1992 ▸) followed by averaging and integration of Bragg peaks using a Monte Carlo integration algorithm (Kirian *et al.*, 2011 ▸). The data-collection statistics are summarized in Supplementary Table S1. Data for MT_1_–Ago-2-PMT were processed as described previously (Stauch *et al.*, 2019 ▸), solving the indexing ambiguity resulting from space group *P*42_1_2 and the very similar lengths of the axes: *c* ≃ *a* = *b*.

### 2.5. Structure determination   

All structures were solved using molecular replacement. The β_2_AR and A_2A_AR data sets were phased using the models of the previously solved structure of β_2_AR bound to timolol (Hanson *et al.*, 2008 ▸; PDB entry 3d4s), the previously solved structure of A_2A_AR bound to ZM241385 (Liu *et al.*, 2012 ▸; PDB entry 4eiy) and the previously solved structure of MT_1_ bound to agomelatine (Stauch *et al.*, 2019 ▸; PDB entry 6me5), respectively, which had all heteroatoms removed from the search model. The structures were further optimized by iterative cycles of rebuilding in *Coot* (Emsley & Cowtan, 2004 ▸) and refinement in *phenix.refine* (Adams *et al.*, 2010 ▸) or *REFMAC*5 (Murshudov *et al.*, 2011 ▸) in the case of MT_1_–Ago-2-PMT. After the receptor refinement had converged, the respective ligand was inserted into the electron density inside the ligand-binding pocket. The final refinement runs were performed with *BUSTER* v.2.10.2 (Smart *et al.*, 2012 ▸) for the β_2_AR structures, *phenix.refine* for A_2A_AR–LUF-ZM and *REFMAC*5 in the case of MT_1_–Ago-2-PMT (Supplementary Tables S1 and S3).

The atomic coordinates and structure factors have been deposited in the Protein Data Bank under the following accession codes (see Table 1 ▸ for an explanation of the data-set names): 6prz (β_2_AR–Alp-Alp), 6ps0 (β_2_AR–Alp-Cara), 6ps1 (β_2_AR–Alp-Tim), 6ps2 (β_2_AR–Tim-Alp), 6ps3 (β_2_AR–Tim-Carv), 6ps4 (β_2_AR–Tim-ICI), 6ps5 (β_2_AR–Tim-Prop), 6ps6 (β_2_AR–Tim-Tim), 6ps7 (A_2A_AR–LUF-ZM) and 6ps8 (MT_1_–Ago-2-PMT).

## 3. Results   

### 3.1. Development of the Complex-LCP method   

The typical GPCR structure-determination process consists of several major steps, including construct design, expression of the target receptor in a heterologous system, purification of the protein in the presence of a stabilizing ligand and crystallization in LCP (Stevens *et al.*, 2013 ▸). The ligand of interest is usually added before receptor solubilization and is continuously supplied throughout purification and crystallization. Following this traditional structure-determination approach, each receptor–ligand combination requires individual screening and optimization of purification and, more importantly, crystallization conditions, which may take weeks to months. Thus, SBDD studies demand substantial efforts proportional to the number of compounds being investigated.

Recently, a more efficient method for multiple GPCR co-crystal structure determination was introduced (Rucktooa *et al.*, 2018 ▸) by crystallizing the target receptor in complex with a low-affinity ‘carrier’ ligand and subsequent soaking of crystals in solutions containing the desired higher affinity compounds. This approach, however, has several limitations. Firstly, it relies on the availability of relatively large high-quality crystals of the receptor in a complex with a low-affinity ligand, which is often challenging as such ligands typically do not sufficiently stabilize the receptors. Secondly, inhomogeneous ligand exchange in a large crystal could potentially disrupt its structure and integrity, leading to a loss of diffraction quality and dissolution of the entire crystal. These difficulties can be overcome by using a receptor thermally stabilized in a specific pre-defined conformation, such as, for example, the adenosine A_2A_ receptor stabilized in an inactive state by nine mutations using the STaR technology (Doré *et al.*, 2011 ▸).

We approached these challenges from a different angle, taking advantage of the LCP-SFX approach (Liu *et al.*, 2013 ▸) that has proven to be highly successful for GPCR structure determination using micrometre-sized crystals grown in LCP (Stauch & Cherezov, 2018 ▸). As a model system, we have selected the human β_2_-adrenergic receptor (β_2_AR), which is one of the most extensively studied GPCRs to date and has a large set of pharmacologically and structurally diverse ligands, including approved drugs such as beta blockers (β_2_AR antagonists) and anti-asthmatic medicines (β_2_AR agonists).

We modified our XFEL sample-preparation protocol (Liu *et al.*, 2014 ▸) by introducing a transient ligand. The function of this ligand is to stabilize the receptor during purification and to enable its crystallization. A suitable transient ligand should have an off-rate that is fast compared with the crystallization timescale in order to facilitate its exchange to the ligand of interest. Therefore, in our Complex-LCP approach the target receptor is first purified in the presence of a minimal amount of the transient ligand and reconstituted in LCP. Concurrent ligand exchange and crystallization is then initiated by overlaying a protein-laden LCP bolus with precipitant solution containing a large excess of the ligand of interest. Dozens of different ligands can be tested in parallel using a single batch (∼1 mg) of the purified receptor reconstituted in LCP. The transient ligand is replaced during the process, leading to co-crystals of the receptor in complex with the desired ligands. Finally, SFX data sets are collected for each ligand of interest and structures are solved using molecular replacement.

### 3.2. Implementation of the protocol   

**Figure 1:**
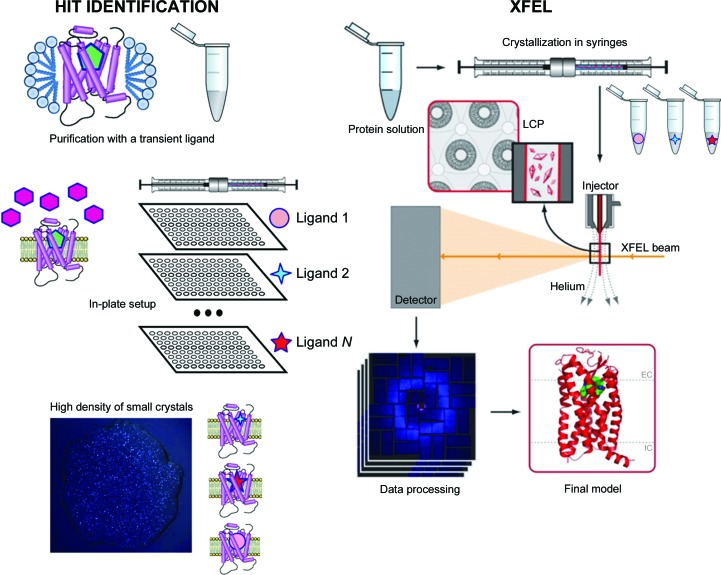
Schematic diagram of the Complex-LCP method. After crystallization conditions have been identified and optimized, the target receptor is purified in complex with a transient ligand and screened against a panel of *N* ligands using nanolitre-volume high-throughput robotic crystallization in 96-well glass sandwich plates. Those ligands that support crystallization are then used to prepare samples for XFEL data collection in syringes.

An overall scheme for the Complex-LCP method is shown in Fig. 1 ▸. In a proof-of-concept study, we used β_2_AR fused to T4 lysozyme (β_2_AR-T4L) and aimed at obtaining structures in complex with eight ligands with different MoAs ranging from inverse agonism to agonism (Table 2 ▸). Two of these ligands (alprenolol and timolol) were selected to play the role of a transient ligand owing to their favorable kinetic properties (Supplementary Table S1). Initial high-throughput crystallization trials were performed in 96-well glass sandwich LCP plates to identify suitable crystallization conditions that would produce showers of small crystals of the receptor purified with each of the two transient ligands. Both ligands were used at a 50 µ*M* concentration in the purified protein sample. In the case of timolol no supplementation of ligand to the crystallization screen was necessary, whereas in the case of alprenolol the screens were supplemented with 100 µ*M* ligand to maintain crystal growth. Following these trials, a condition based on ammonium sulfate as a precipitant salt was chosen for further steps (100 m*M* ammonium sulfate, 100 m*M* HEPES pH 7.0, 30% PEG 400, 1 m*M* ligand).

Next, the receptor was purified in the presence of 50 µ*M* of the transient ligand (alprenolol or timolol), and LCP crystallization trials were set up in 96-well glass sandwich plates using the precipitant solution supplemented with 1 m*M* of the target ligand, with one plate per ligand. Two additional control plates were also set up: Control Plate A without any ligand in the precipitant solutions (negative control) and Control Plate B with the same transient ligand as that used during purification added to the precipitant solutions (positive control). Crystallization plates were stored at 20°C in a RockImager 1000 (Formulatrix) and were inspected at 12 h intervals.

On the first day after setup, showers of small crystals appeared in most wells of all plates with ligands added to the precipitant solutions (Supplementary Fig. S1). On the second day, however, crystals in plates with the agonists formoterol and procaterol started to dissolve, and they completely disappeared on day 3 [Supplementary Fig. S1(*b*)]. We interpreted this phenomenon as an indication that agonist binding induces a substantially different receptor conformation that is incompatible with pre-formed crystal contacts. Crystals in plates with the remaining six ligands reached a maximal size of up to 5–10 µm and remained stable until the end of the observation period of one month. As expected, the same behavior was observed in Control Plate B, while no crystals or only very small (∼1 µm) and sparse crystals were found in Control Plate A.

After confirming crystal formation in plates, the crystallization volume was scaled up ∼100 times in Hamilton gas-tight syringes for LCP-SFX data collection, following our previously developed protocol (Liu *et al.*, 2014 ▸). Owing to the large excess of precipitant solution over LCP used in the crystallization setup, the concentration of the transient ligand decreases 50 times upon equilibration, resulting in a ∼1000:1 molar excess of each of the target ligands over the transient ligand to ensure efficient ligand exchange.

### 3.3. LCP-SFX data collection   

LCP-SFX data were collected as described previously (Liu *et al.*, 2013 ▸), with microcrystals delivered to the XFEL beam within their crystal-growth medium by an LCP injector (Weierstall *et al.*, 2014 ▸). Except for two data sets (Table 1 ▸), the data were acquired at the CXI experimental station (Boutet & Williams, 2010 ▸) of LCLS using a vacuum sample chamber with the XFEL beam focused to about 1.5 µm diameter and the CSPAD (Hart *et al.*, 2012 ▸), operating at an XFEL pulse repetition rate of 120 Hz. Data for carvedilol and propranolol were collected at SACLA (Ishikawa *et al.*, 2012 ▸) using a helium-filled sample chamber (Kameshima *et al.*, 2014 ▸), a 1.5 µm diameter beam size and an MPCCD detector (Tono *et al.*, 2015 ▸), operating at an XFEL pulse repetition rate of 30 Hz. Crystals were delivered using an LCP injector with a 50 µm inner diameter (ID) nozzle and a flow rate of 150–200 nl min^−1^ for experiments at LCLS and with a 100 µm ID nozzle and a flow rate of 250 nl min^−1^ at SACLA. All acquired LCP-SFX data were processed using a Monte Carlo integration approach implemented in *CrystFEL* (White *et al.*, 2016 ▸), which requires a large number of indexed patterns for the accurate determination of intensities. In our experience, 20 000–30 000 indexed images are sufficient to assemble a high-quality data set. However, when the overall receptor structure is known and only the binding pose of the ligand is being determined, fewer indexed images, of the order of 5000–10 000, may be sufficient (Table 1 ▸). With the parameters described above and a crystal hit/indexing rate of >1%, it is currently possible to collect a complete data set at LCLS within less than 2 h using about 25 µl of crystal-laden LCP.

### 3.4. Ligand electron-density maps and structure validation   

**Figure 2:**
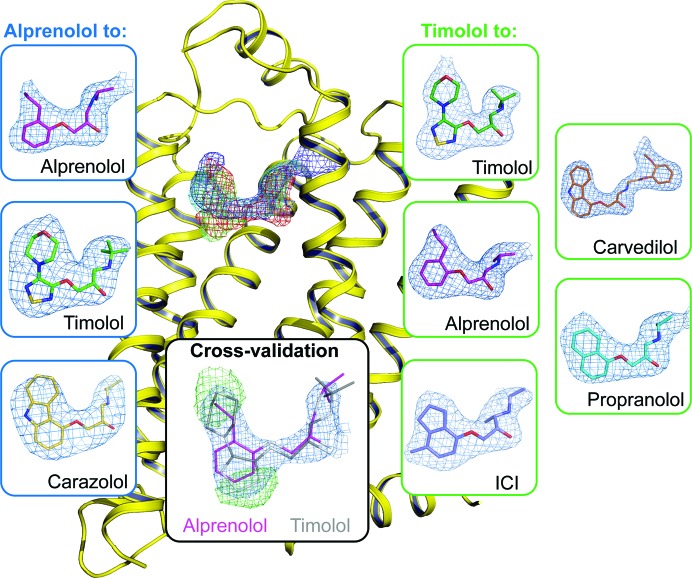
Electron densities for the β_2_AR ligands obtained using the Complex-LCP method. 2*mF*_o_ − *DF*_c_ electron densities in the ligand-binding pocket (purple circle) right after molecular replacement are shown as superimposed meshes contoured at 0.7σ (timolol, green; alprenolol, red; carvedilol, blue; propranolol, cyan). The *mF*_o_ − *DF*_c_ polder ligand OMIT maps (Liebschner *et al.*, 2017 ▸) contoured at 3σ are shown for each ligand inside blue (transient ligand alprenolol) and green (transient ligand timolol) boxes. The inset in the black box demonstrates cross-validation of the ligand exchange using crystallographic data. When alprenolol-to-timolol exchange data are refined with alprenolol, the residual *mF*_o_ − *DF*_c_ electron density (green) contoured at 3σ clearly indicates that the transient ligand has been successfully exchanged.

Successful molecular replacement using the known β_2_AR structure immediately revealed strong *mF*_o_ − *DF*_c_ electron densities in the ligand-binding pocket resembling the shapes of the target ligands (Fig. 2 ▸). To further validate the ligand exchange, we refined the coordinates after placing the corresponding transient ligand in the density. The resulting *mF*_o_ − *DF*_c_ maps showed substantial positive and negative electron density around the ligand (Fig. 2 ▸ and Supplementary Fig. S2), indicating that the presence of the transient ligand in the complex is not supported by the experimental data and thus confirming that the transient ligand has successfully exchanged during the process of crystallization. Conversely, the refinement of structures against the experimental data after placing the corresponding target ligands in the density produced well defined maps fully consistent with the chemical structures of the ligands used. The final data-processing and refinement statistics are shown in Supplementary Table S2.

### 3.5. Comparison of synchrotron and XFEL structures   

The XFEL β_2_AR structures in complex with carazolol, timolol, alprenolol and ICI-118,551 obtained in this work are almost identical to those previously determined using synchrotron data collected from cryocooled crystals (Supplementary Table S3; Cherezov *et al.*, 2007 ▸; Wacker *et al.*, 2010 ▸; Hanson *et al.*, 2008 ▸), validating our approach. The resolution cutoff values of the data sets range from 2.4 Å for alprenolol to 3.4 Å for carazolol. The variation in data quality most likely arises from a combination of factors such as crystal quality for the particular ligand and the number of indexed images for a given data set. Crystal quality also appears to depend on the identity of the transient ligand used for the exchange: the resolution of the structures based on alprenolol as a transient ligand is consistently lower compared with those based on timolol. This observation can be explained by potentially stronger crystal contacts in the initial seed crystals in the complex with timolol; however, it requires further investigation. The mean *B* factor of the obtained structures correlates with resolution (Supplementary Fig. S3) and on average is ∼30 Å^2^ higher than the mean *B* factor of the corresponding synchrotron structures, reflecting the differences in data-collection temperature and data processing.

### 3.6. Structures of β_2_AR bound to carvedilol and propranolol   

In addition to the four ligands that have previously been co-crystallized with β_2_AR (Wacker *et al.*, 2010 ▸; Cherezov *et al.*, 2007 ▸; Hanson *et al.*, 2008 ▸), we used the Complex-LCP method to determine two new β_2_AR structures in complex with carvedilol and propranolol. Both of these ligands belong to the class of beta blockers; however, carvedilol acts as a β-arrestin-biased agonist, while concomitantly antagonizing G protein activity (Drake *et al.*, 2008 ▸). This characteristic of carvedilol has important pharmacological implications owing to its improved cardioprotective effects compared with the majority of current beta blockers (Leonetti & Egan, 2012 ▸).

**Figure 3:**
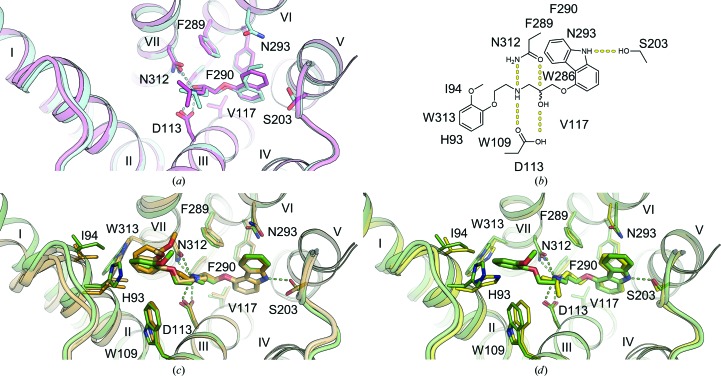
Binding of propranolol and carvedilol to β_2_AR. (*a*) Superposition of propranolol-bound (cyan) and alprenolol-bound (pink) β_2_AR structures. (*b*) Chemical structure of carvedilol with contacting β_2_AR residues within 4 Å of the ligand. (*c*) Superposition of carvedilol-bound β_2_AR (green) and β_1_AR (orange; PDB entry 4amj; two molecules from one asymmetric unit; Warne *et al.*, 2012) structures. (*d*) Superposition of carvedilol-bound (green) and carazolol-bound (yellow) β_2_AR structures. Hydrogen bonds are shown as dashed lines for the propranolol- and carvedilol-bound β_2_AR structures only. Helices are labeled with Roman numerals.

The propranolol-bound β_2_AR structure reveals a canonical ligand-binding pose with the ethanolamine moiety occupying the same position and engaging in hydrogen bonds to Asp113^3.32^ and Asn312^7.39^ (where the superscripts refer to the Ballesteros–Weinstein Class A GPCR numbering scheme; Ballesteros & Weinstein, 1995 ▸), as in the structures with other antagonists (for example alprenolol), and with the naphthalene ring reinforcing the hydrophobic interactions [Fig. 3 ▸(*a*)].

Carvedilol also binds β_2_AR in a similar pose, anchored by Asp113^3.32^ and Asn312^7.39^, with the terminal methoxybenzene group of its tail, which is believed to be responsible for the β-arrestin-biased activity of carvedilol, participating in hydrophobic interactions with His93^2.64^, Ile94^2.65^, Trp109^3.28^ and Trp313^7.40^ [Fig. 3 ▸(*b*)]. Superposition of our human β_2_AR–carvedilol structure with the structure of turkey β_1_AR–carvedilol reported previously using the receptor stabilized by eight point mutations (Warne *et al.*, 2012 ▸) reveals several notable differences [Fig. 3 ▸(*c*)]. While the ligands overlap well in the carbazole head and the oxypropanolamine tail (r.m.s.d. of 0.45 Å) common to many β_2_AR ligands (Hanson *et al.*, 2008 ▸; Cherezov *et al.*, 2007 ▸; Wacker *et al.*, 2010 ▸), the terminal methoxybenzene group adopts slightly different orientations in these two receptors. Comparing the structures of the receptors, we observe a 1–2 Å outward tilt of the extracellular tips of helices II and VII in β_1_AR. The extra space in the β_1_AR pocket created by these displacements potentially allows a more dynamic conformation of the carvedilol tail (different orientations in the two molecules in the asymmetric unit) compared with β_2_AR. Additionally, the extracellular tip of helix I that does not interact with the ligand is also shifted ∼3 Å between these structures, which may reflect differences between these receptors and/or differences in their crystallization environment and crystal packing.

A comparison of carvedilol- and carazalol-bound β_2_AR structures shows displacements of several residues interacting with the methoxybenzene group of carvedilol, leading to an ∼0.6 Å outward shift of helix II and extracellular loop 2 (ECL2) and expanding the volume of the ligand-binding pocket [Fig. 3 ▸(*d*)]. These small structural differences may be responsible for the differences in the reported ligand MoA.

### 3.7. Application of the Complex-LCP method to other receptors   

**Figure 4:**
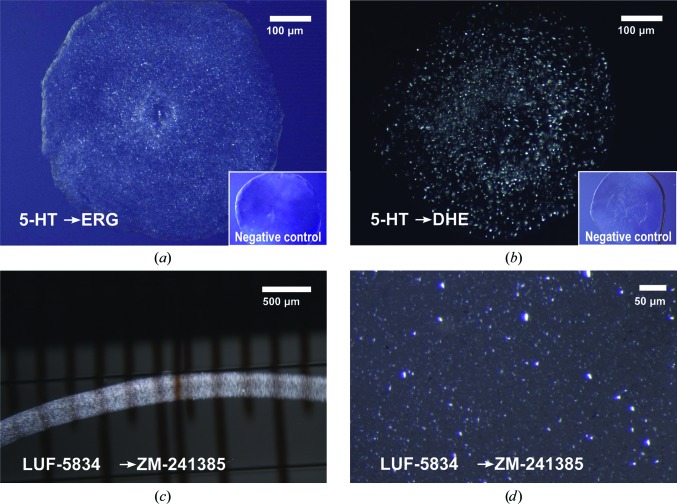
Ligand exchange in 5-HT_2B_ and A_2A_AR. (*a*, *b*) Ligand-exchange experiment in LCP crystallization plates with 5-HT_2B_. The transient ligand serotonin was exchanged for ERG (*a*) and DHE (*b*). The images were taken under cross-polarized light. No crystals appeared in control experiments (insets) set up without adding ligands to the precipitant solution. (*c*, *d*) Ligand-exchange experiment in glass syringes with A_2A_AR. The transient ligand LUF5834 is exchanged for ZM241385. (*c*) LCP string immersed in precipitant solution in a glass syringe. (*d*) LCP sample titrated with 7.9 MAG before loading into an LCP injector.

To demonstrate the general applicability of the described Complex-LCP method, we applied it to four other GPCRs: serotonin receptors 1B (5-HT_1B_) and 2B (5-HT_2B_), adenosine A_2A_ receptor (A_2A_AR) and melatonin receptor type 1A (MT_1_). An overview of all of the compounds used as transient or target ligands in this study can be found in Supplementary Fig. S4. At the time these experiments were performed, only two agonists, ergotamine (ERG) and dihydroergotamine (DHE), had been co-crystallized with serotonin receptors (Wacker *et al.*, 2013 ▸; Wang *et al.*, 2013 ▸); therefore, one of them, ERG, was initially chosen as a transient ligand. Microcrystals of 5-HT_1B_ were generated following the same procedure as for β_2_AR with the goal of exchanging ERG for the antagonist methylergometrine or for the agonists oxymetazoline, sumatripan and RU-24969 (Supplementary Fig. S4). However, after collecting diffraction data and solving the structures it was observed that the ligand exchange had been unsuccessful and that the receptor was still bound to the transient ligand ERG. This result emphasizes the importance of proper transient ligand selection. ERG is a relatively large molecule with multiple interactions within the binding pocket of 5-HT_1B_, leading to a slow off-rate (*k*_off_ = 0.0125 min^−1^; Unett *et al.*, 2013 ▸), which is likely to explain its low exchange efficiency. After realizing the need for a transient ligand with a high off-rate, we switched to serotonin (*k*_off_ = 0.1216 min^−1^; Unett *et al.*, 2013 ▸) and performed crystallization experiments with 5-HT_2B_, exchanging serotonin for ERG or DHE. Microcrystals were obtained in both cases within one day [Figs. 4 ▸(*a*) and 4 ▸(*b*)], while no crystals were observed in the control experiment without any ligand supplemented in the precipitant.

A_2A_AR is another prototypical GPCR, which similarly to β_2_AR has a large number of available ligands. A_2A_AR has been crystallized in complex with many different ligands, including the relatively large antagonists ZM241385 (Liu *et al.*, 2012 ▸) and comp-1 (Sun *et al.*, 2017 ▸), the agonist UK-432097 (Xu, Wu *et al.*, 2011 ▸) and some smaller, high-off-rate antagonists such as XAC, caffeine, PSB36 and theophylline (Cheng *et al.*, 2017 ▸; Doré *et al.*, 2011 ▸). The latter structures with xanthines could only be obtained using a thermostabilized construct of A_2A_AR. We applied our approach using the antagonist LUF5834 (Supplementary Fig. S4) as a transient ligand, successfully exchanging it for the higher affinity antagonist ZM241385 [Figs. 4 ▸(*c*) and 4 ▸(*d*)], and solved the co-crystal structure by SFX (Supplementary Table S4 and Supplementary Fig. S5). As expected, the data quality and the overall A_2A_AR–LUF-ZM structure derived via the Complex-LCP method are nearly identical (the C^α^ r.m.s.d. with PDB entry 5k2d is 0.21 Å) to published A_2A_AR–ZM241385 structures obtained using conventional LCP crystallization (Batyuk *et al.*, 2016 ▸; Liu *et al.*, 2012 ▸).

**Figure 5:**
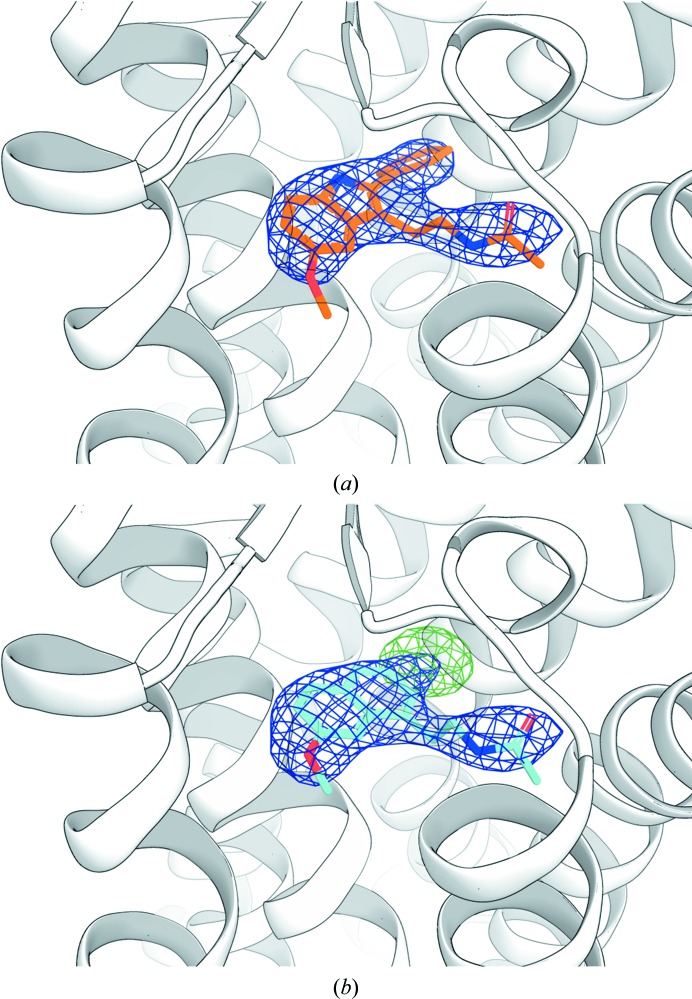
Electron density in the MT_1_ ligand-binding pocket. (*a*) Ligand electron density for MT_1_ (white cartoon) after ligand exchange of agomelatine (Ago) to 2-phenylmelatonin (2-PMT; orange). (*b*) Ligand electron density after incorrect placement of the transient ligand Ago (cyan) and refinement starting from the same molecular-replacement structure (see Section 2). Both structural models are of comparable stereochemical quality, but the structure refined with Ago shows slightly worse refinement statistics (*R* and *R*_free_ of 0.257 and 0.314 compared with 0.257 and 0.308, respectively). 2*mF*_o_ − *DF*_c_ maps (blue mesh) are contoured at 1σ. *mF*_o_ − *DF*_c_ electron-density difference maps contoured at ±3.5σ (green and red for positive and negative peaks, respectively) show strong (∼6.7σ) positive difference density for the missing phenyl ring in the structure refined with Ago.

Melatonin receptor MT_1_ has recently been co-crystallized with several agonists, including two melatonin analogs as well as two drugs: the sleeping aid ramelteon and the atypical antidepressant agomelatine (Stauch *et al.*, 2019 ▸). Since addition of the ligand to the precipitant solution was essential for crystallization in all cases, indicating that ligand-exchange events happen on a timescale comparable to crystallization, we expected MT_1_ to be a suitable target for our Complex-LCP method. We used the antidepressant agomelatine (Ago) as a transient ligand during crystallization setups and successfully exchanged it for the larger 2-phenylmelatonin (2-PMT), as unambiguously identified in the resulting electron densities (Supplementary Fig. S4 and Fig. 5 ▸). The size and diffraction quality of the resulting MT_1_–Ago-2-PMT crystals were comparable with those of published crystals of MT_1_–Ago (PDB entry 6me5; Stauch *et al.*, 2019 ▸), suggesting that the transient ligand (in this case Ago) was the limiting factor to the final resolution. Again, as in the case with A_2A_AR, our MT_1_–Ago-2-PMT structure is nearly identical to the published structure of MT_1_–2-PMT (the C^α^ r.m.s.d. with PDB entry 6me3 is 0.49 Å), microcrystals of which were obtained using conventional LCP crystallization (Stauch *et al.*, 2019 ▸).

## 4. Discussion   

We have introduced a new method, Complex-LCP, for facilitating the structure determination of multiple GPCR–ligand complexes. The method allows the rapid identification of ligand-binding poses and interactions for a panel of about ten ligands in a single experiment. The two most critical aspects of this method are the use of a transient ligand to increase the stability and conformational homogeneity of the target receptor and the application of an XFEL source for crystallographic data collection from micrometre-sized crystals. Several considerations were deemed to be essential for the selection of the transient ligand, such as a fast ligand off-rate to ensure efficient ligand exchange and a relatively low dissociation constant, *K*_d_, compared with the ligands of interest to maintain a high target ligand/transient ligand concentration ratio during ligand exchange. As conceived, ligand exchange can occur at different stages of the process: before, during or after crystal nucleation. For example, in the case of A_2A_AR the transient ligand LUF5834 is likely to be exchanged with ZM241385 before crystallization, as no crystals were obtained when using LUF5834 alone. On the other hand, in the case of β_2_AR the transient ligand is apparently involved in crystal nucleation and is replaced after the crystals have already formed. This conclusion is supported by the observation of crystal dissolution in the presence of the agonists procaterol and formoterol and by the prominent effect of the transient ligand on the resolution of the obtained structures, so that using timolol rather than alprenolol as the transient ligand resulted in higher resolution structures (Table 1 ▸). Most strikingly, exchanging timolol for alprenolol substantially improved the resolution (β_2_AR–Tim-Alp data set, 2.4 Å resolution) compared with the control sample using alprenolol alone (β_2_AR–Alp-Alp data set, 2.8 Å resolution) and with the previously reported β_2_AR–alprenolol synchrotron structure (PDB entry 3nya, 3.16 Å resolution; Wacker *et al.*, 2010 ▸). It is evident that such improvements in resolution are highly desirable for SBDD applications.

The availability of a protein construct with an adequate conformational stability to generate crystals is a prerequisite for the Complex-LCP method. The method has not been devised to provide an alternative to thermostabilization by point mutations, the introduction of which might still be necessary for crystallogenesis. Indeed, most of the GPCR constructs used for successful crystallization in this study and elsewhere (Xiang *et al.*, 2016 ▸) contained one or more point mutations (see Section 2 for details). Compounds with fast off-rates that are potential candidates for the role of transient ligands are often less stabilizing than super-high-affinity ligands and may require additional receptor engineering for crystallization.

The Complex-LCP method relies on the ability to collect high-resolution crystallographic data from micrometre-sized crystals, which is enabled by the SFX approach at XFEL sources. The small size of the microcrystals facilitates ligand exchange without affecting the crystal quality and integrity. Additionally, the lower mosaicity and the fewer growth defects in microcrystals compared with their larger counterparts used for data collection at synchrotron sources, as well as bypassing crystal harvesting and potential artifacts from cryocooling, often lead to higher quality diffraction, as demonstrated by several examples (Fenalti *et al.*, 2015 ▸; Zhang *et al.*, 2015 ▸; Kang *et al.*, 2015 ▸). Recent advances in SFX data-processing algorithms and XFEL sample-delivery instrumentation have reduced the requirements for the amount of crystalline sample per data set. Further optimizations and improvements are imminent, such as fixed-target-based crystal delivery (Roedig *et al.*, 2017 ▸; Mueller *et al.*, 2015 ▸; Hunter *et al.*, 2015 ▸) combined with higher repetition-rate XFEL sources, which could substantially increase the throughput of this method.

While this approach and its variations have a strong potential to accelerate drug-discovery applications using difficult-to-crystallize membrane proteins, their broader acceptance by academic and industrial laboratories may be limited by the shortage of available XFEL beamtime. Recent demonstrations of serial crystallography at synchrotron sources (Martin-Garcia *et al.*, 2017 ▸; Meents *et al.*, 2017 ▸; Weinert *et al.*, 2017 ▸) indicate that new-generation diffraction-limited storage rings and other related developments (Eberhardt, 2015 ▸; Yabashi & Tanaka, 2017 ▸) promise to deliver crystallographic data from micrometre-sized crystals that are comparable in quality to those from XFELs. We believe that these advancements will produce a strong impact on the development of more efficient and safe therapies.

## 5. Related literature   

The following reference is cited in the supporting information for this article: Sykes *et al.* (2014 ▸).

## Supplementary Material

PDB reference: β_2_-adrenergic receptor, complex with alprenolol, 6prz

PDB reference: 6ps2

PDB reference: complex with carazolol, 6ps0

PDB reference: complex with timolol, 6ps1

PDB reference: 6ps6

PDB reference: complex with carvedilol, 6ps3

PDB reference: complex with ICI-118,551, 6ps4

PDB reference: complex with propranolol, 6ps5

PDB reference: adenosine A_2A_ receptor, complex with ZM241385, 6ps7

PDB reference: melatonin receptor MT_1_, complex with 2-phenylmelatonin, 6ps8

Supplementary Tables and Figures. DOI: 10.1107/S2052252519013137/cw5024sup1.pdf

## Figures and Tables

**Table 1:** XFEL data-collection statistics The names of the data sets contain the transient ligand followed by the ligand of interest. Alp, alprenolol; Tim, timolol; Cara, carazolol; Carv, carvedilol; ICI, ICI-118,551; Prop, propanolol; LUF, LUF5834; ZM, ZM241385; Ago, agomelatine; 2-PMT, 2-phenylmelatonin.

Data set	XFEL	Data-collection time (min)	Total No. of images	No. of hits	Hit rate (%)	No. of indexed images	Indexing rate (%)	Resolution at CC* = 0.5† (Å)
β_2_AR–Alp-Alp	LCLS	195	1405887	149024	10.6	39599	26.6	2.8
β_2_AR–Alp-Tim	LCLS	209	1503057	238986	15.9	59814	25.0	3.2
β_2_AR–Alp-Cara	LCLS	147	1061949	41416	3.9	9493	22.9	3.4
β_2_AR–Tim-Alp	LCLS	108	775070	99984	12.9	60694	60.7	2.4
β_2_AR–Tim-Carv	SACLA	33	59426	28465	47.9	14579	51.2	2.5
β_2_AR–Tim-ICI	LCLS	103	739335	116815	15.8	43660	37.4	2.6
β_2_AR–Tim-Tim	LCLS	128	918091	80792	8.8	17952	22.2	2.7
β_2_AR–Tim-Prop	SACLA	65	117972	8494	7.2	5201	61.2	2.9
A_2A_AR–LUF-ZM	LCLS	46	241932	68623	28.4	39281	57.2	1.85
MT_1_–Ago-2-PMT	LCLS	136	977748	87453	8.9	65260	74.6	3.3

† The reported resolution may depend on the number of indexed patterns used for each data set.

**Table 2:** β_2_AR ligands used in the exchange experiments with their MoAs, molecular weights (MW) and affinity (*K*_i_) values Data are from the ChEMBL database (ChEMBL_23; Gaulton *et al.*, 2012 ▸). Chemical structures of the ligands used for the other receptors in this study are shown in Supplementary Fig. S4.

Ligand	Chemical structure	MoA	ChEMBL ID	MW (Da)	*K*_i_ (n*M*)	Reference
ICI-118,551	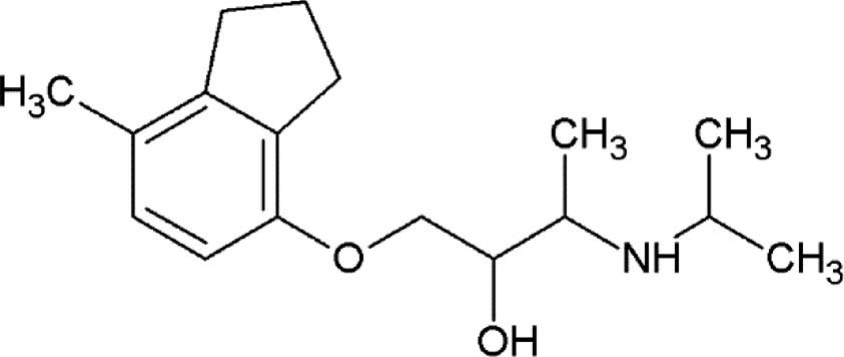	Inverse agonist	CHEMBL513389	277.4	0.13	Dolušić *et al.* (2011 ▸)
Carazolol	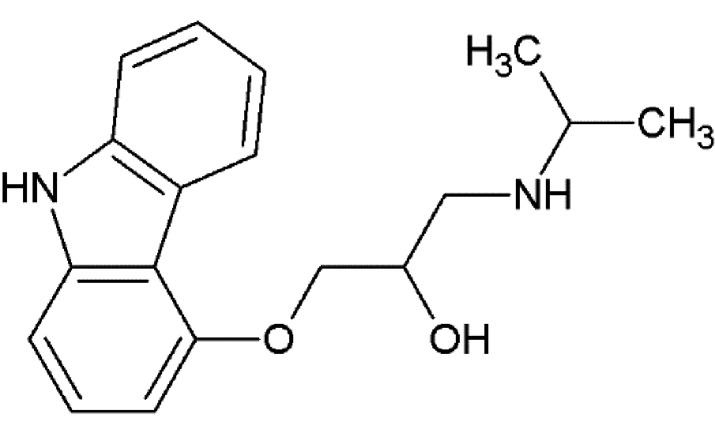	Inverse agonist	CHEMBL324665	298.4	0.114	Sabio *et al.* (2008 ▸)
Timolol	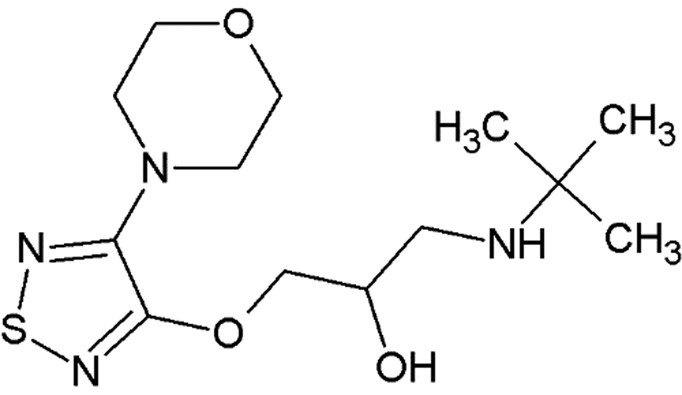	Inverse agonist	CHEMBL499	316.4	0.201†	
Propranolol	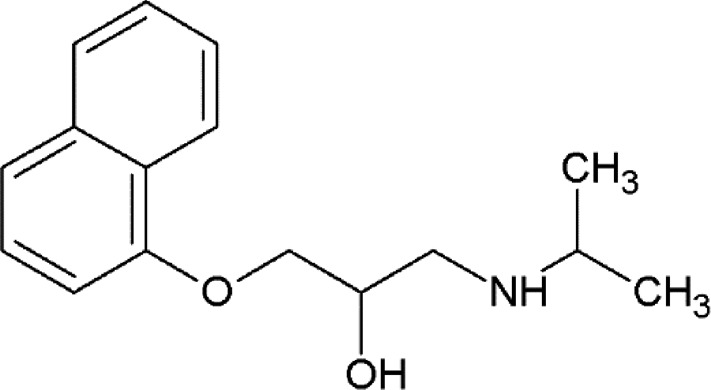	Inverse agonist	CHEMBL27	259.3	3.69	Plazinska *et al.* (2014 ▸)
Alprenolol	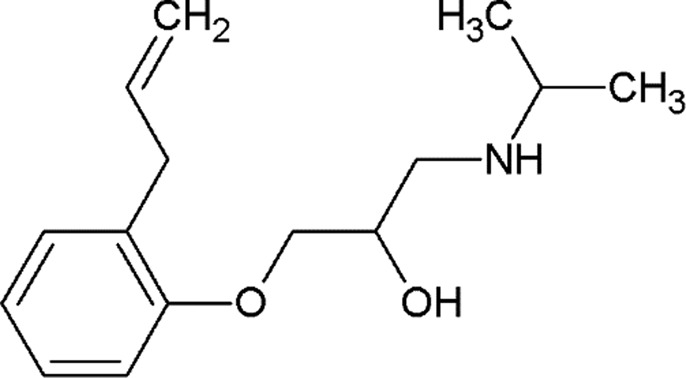	Antagonist	CHEMBL266195	249.4	1	Aristotelous *et al.* (2013 ▸)
Carvedilol	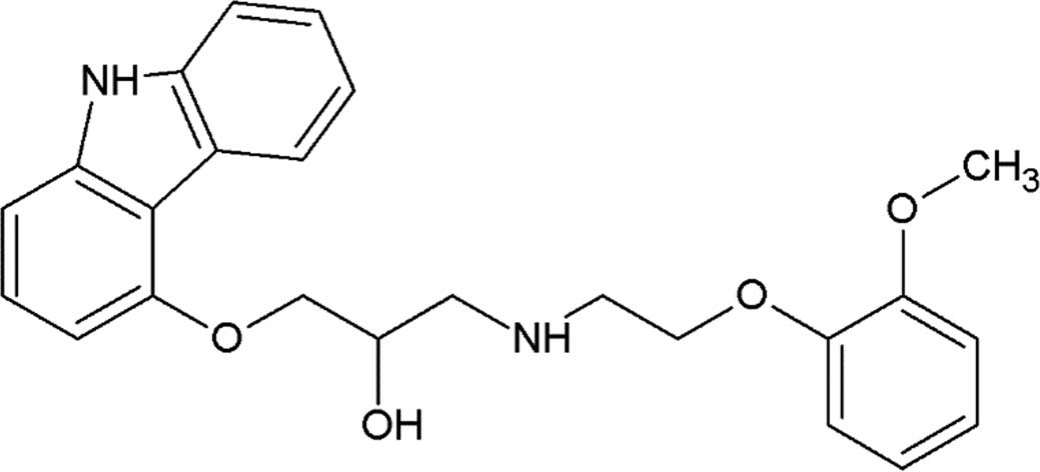	β-Arrestin-biased agonist	CHEMBL723	406.5	0.166†	
Procaterol	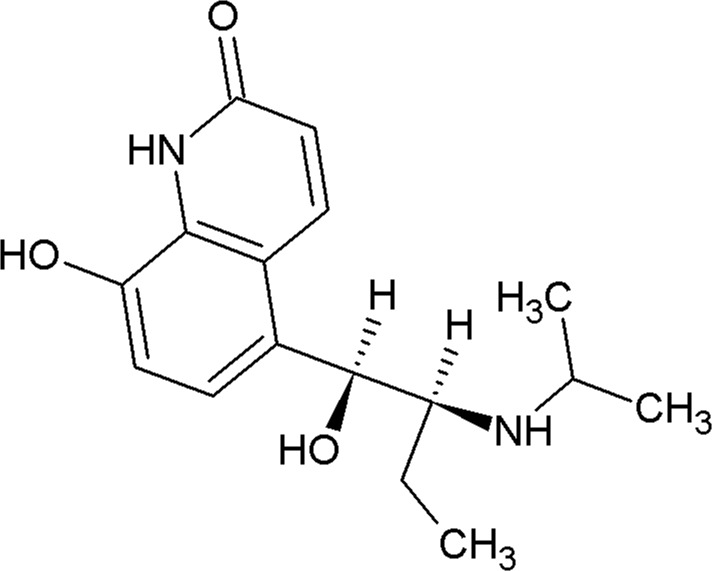	Agonist	CHEMBL160519	290.4	78	Baker (2010 ▸)
Formoterol	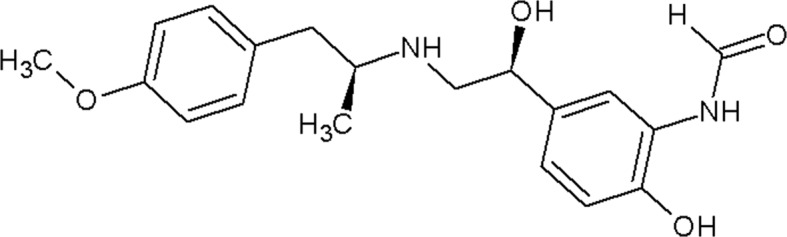	Agonist	CHEMBL3989798	344.4	23	Baker (2010 ▸)

† Values are from the DrugMatrix Database (https://ntp.niehs.nih.gov/drugmatrix/index.html).
