# Supplementary material for: Toward G protein-coupled receptor structure-based drug design using X-ray lasers
Source: IUCrJ. 2019 Oct 24;6(Pt 6):1106–19. doi: 10.1107/S2052252519013137 (PMC6830214; doi:10.1107/S2052252519013137)
Supplement: Supplementary file 1 [file m-06-01106-sup1.pdf]

IUCrJ

Volume 6 (2019)

Supporting information for article:

Toward G protein-coupled receptor structure-based drug design using X-ray lasers

Andrii Ishchenko, Benjamin Stauch, Gye Won Han, Alexander Batyuk, Anna Shiriaeva, Chufeng Li, Nadia Zatsepin, Uwe Weierstall, Wei Liu, Eriko Nango, Takanori Nakane, Rie Tanaka, Kensuke Tono, Yasumasa Joti, So Iwata, Isabel Moraes, Cornelius Gati and Vadim Cherezov

Table S1 Kinetic properties of transient ligands used for Complex-LCP experiments in β_2AR.

Ligand	Structure	MoA	CHEMBL ID	k_{on} ($M^{-1}s^{-1}$)	k_{off} (s^{-1})	K_D (nM)	Reference
timolol	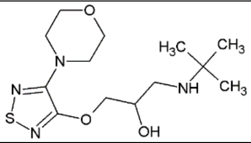	inverse agonist	CHEMBL499	62.67×10^5	5.5×10^{-4}	0.09	(Sykes *et al.*, 2014)
alprenolol	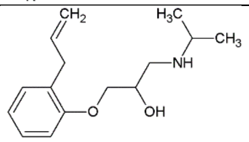	antagonist	CHEMBL266195	8.11×10^5	6.4×10^{-4}	0.79	(Aristotelous *et al.*, 2013)

Table S2 Data collection and refinement statistics for β_2AR.

Receptor	β_2AR							
Ligand<br>PDB entry	Alp-Alp<br>6prz	Alp-Cara<br>6ps0	Alp-Tim<br>6ps1	Tim-Alp<br>6ps2	Tim-Carv<br>6ps3	Tim-ICI<br>6ps4	Tim-Prop<br>6ps5	Tim-Tim<br>6ps6
**Data collection**								
No. indexed frames	39,599	9,493	59,814	60,694	14,579	43,660	5,201	17,952
Space group	$P2_12_12_1$	$P2_12_12_1$	$P2_12_12$	$P2_12_12_1$	$P2_12_12_1$	$P2_12_12_1$	$P2_12_12_1$	$P2_12_12_1$
Cell dimensions, Å								
a	41.8	41.5	41.7	41.7	42.6	41.5	42.7	41.5
b	76.2	75.8	76.3	76.2	77.4	76.2	77.6	76.2
c	171.4	170.4	172.0	171.9	173.9	171.6	173.9	172.0
Resolution, Å	30.9 – 2.8 (3.02 – 2.8)	29.7 – 3.4 (3.8 – 3.4)	30.9 – 3.2 (3.58 – 3.2)	30.8 – 2.4 (2.52 – 2.4)	46.3 – 2.5 (2.63 – 2.5)	37.3 – 2.6 (2.76 – 2.6)	46.4 – 2.9 (3.13 – 2.9)	31.4 – 2.7 (2.89 – 2.7)
No. reflections	4,887,400	2,113,239	6,470,728	11,220,527	5,225,563	5,931,298	1,420,168	2,690,075
No. unique reflections	14,199	7,931	9,646	22,282	20,630	17,521	13,464	15,738
R_{split}, %	15.1 (231.9)	23.6 (163.3)	17.2 (188.4)	8.9 (206.3)	13.8 (282.5)	17.5 (163.4)	23.3 (185.4)	17.7 (185.5)
Mean $I/\sigma(I)$	5.0 (0.48)	4.2 (0.69)	5.7 (0.58)	6.2 (0.51)	4.1 (0.38)	4.7 (0.70)	2.8 (0.57)	4.2 (0.60)
CC^*, %	0.9981 (0.519)	0.9905 (0.694)	0.9988 (0.652)	0.9986 (0.676)	0.9959 (0.518)	0.9975 (0.632)	0.9900 (0.606)	0.9964 (0.628)
Completeness, %	100 (100)	100 (100)	100 (100)	100 (100)	100 (100)	100 (100)	100 (100)	100 (100)
Multiplicity	344.2 (65.0)	266.5 (182.4)	670.8 (78.1)	503.6 (156.9)	253.3 (132.2)	338.5 (32.9)	104.0 (62.1)	170.9 (30.7)
**Refinement**								
Resolution, Å	30.9-2.80	29.7-3.40	30.9-3.20	30.8-2.40	46.3-2.50	37.3-2.60	46.4-2.90	31.4-2.70
No. reflections/free set	14,103/706	7,885/393	9,559/476	22,207/1,110	20,414/1,022	17,428/870	13,417/673	15,898/796
R_{work}/ R_{free}, %	20.7/22.8	20.8/26.0	22.9/26.4	18.1/22.3	20.3/24.1	20.4/24.4	21.0/25.7	20.0/23.3
No. atoms								
Protein	3,506	3,446	3,410	3,577	3,497	3,527	3,466	3,487
Ligand	18	22	21	18	30	20	19	21
Lipids and other	117	83	86	284	186	256	136	215
B-factors, Å^2								
Overall	106.1	118.6	135.1	89.4	95.5	61.9	104.3	86.9
Receptor	101.4	113.1	129.9	84.6	92.0	56.8	99.8	81.9
T4L	112.3	127.3	144.9	93.5	99.1	65.6	111.2	92.5
Ligand	105.5	120.6	134.6	78.1	88.5	58.1	95.7	70.0
Lipids and other	131.0	139.5	163.9	111.5	114.8	87.7	120.4	108.6
R.m.s deviations								
Bond lengths, Å	0.009	0.009	0.009	0.010	0.010	0.009	0.009	0.009
Bond angle, °	0.94	1.01	0.96	1.03	1.06	0.99	1.00	1.05
Ramachandran stats, %								
Favored	96.6	96.1	97.5	96.6	98.2	96.8	98.4	98.6
Allowed	3.4	3.9	2.5	3.4	1.8	3.2	1.6	1.4
Disallowed	0	0	0	0	0	0	0	0

*Values in parentheses are for highest-resolution shell.

Table S3 Root mean square deviations (RMSD) between β_2AR structures solved previously using synchrotron radiation and β_2AR structures obtained in this work, excluding fusion proteins.

**Ligand**		**Synch. structure (reference)**	**RMSD, Å**		
			**Cα atoms**	**All atoms**	**Ligand**
Alprenolol	Alp-Alp	3nya	0.39	0.99	0.80
	Tim-Alp	3nya	0.37	0.89	0.80
Timolol	Tim-Tim	3d4s	0.33	0.85	0.48
	Alp-Tim	3d4s	0.34	0.78	0.52
Carazolol	Alp-Cara	2rh1	0.63	1.11	0.58
ICI 118,551	Tim-ICI	3ny8	0.37	0.75	0.59

Table S4 Data collection and refinement statistics for $A_{2A}AR$ and MT_1.

Receptor	$A_{2A}AR$	MT_1
Ligand	LUF-ZM	Ago-2-PMT
PDB entry	6ps7	6ps8
**Data collection**		
No. indexed frames	39,281	65,260
Space group	$C222_1$	$P42_12$
Cell dimensions, Å		
a	40.3	122.2
b	180.5	122.2
c	142.5	122.6
Resolution, Å	33.5-1.85 (1.90-1.85)	30-3.30 (3.39-3.30)
No. reflections	5,329,234	24,215,540
No. unique reflections	45,163	14,561
$R_{split,}$ %	13.6 (217)	11.9 (344)
Mean $I/\sigma(I)$	4.6 (0.53)	8.5 (0.35)
*CC**, %	0.9956 (0.524)	0.9994 (0.529)
Completeness, %	100 (100)	100 (100)
Multiplicity	118 (51.1)	1,663 (178)
**Refinement**		
Resolution, Å	33.5-1.85	30.0-3.30
No. reflections/free set	45,045/1,136	13,778/728
R_{work}/ R_{free}, %	17.2/19.8	25.8/30.8
No. atoms		
Protein	3,210	3,648
Ligand	25	23
Lipids and other	500	0
B-factors, $Å^2$		
Overall	52.9	114.2
Receptor	39.4	123.0
BRIL or PGS	85.1	119.0
Ligand	31.2	107.4
Lipids and other	68.8	n/a
R.m.s deviations		
Bond lengths, Å	0.007	0.008
Bond angle, °	1.11	1.11
Ramachandran stats, %		
Favored	98.1	94.3
Allowed	1.9	5.7
Disallowed	0	0.2 (Tyr-79)

*Values in parentheses are for highest-resolution shell.

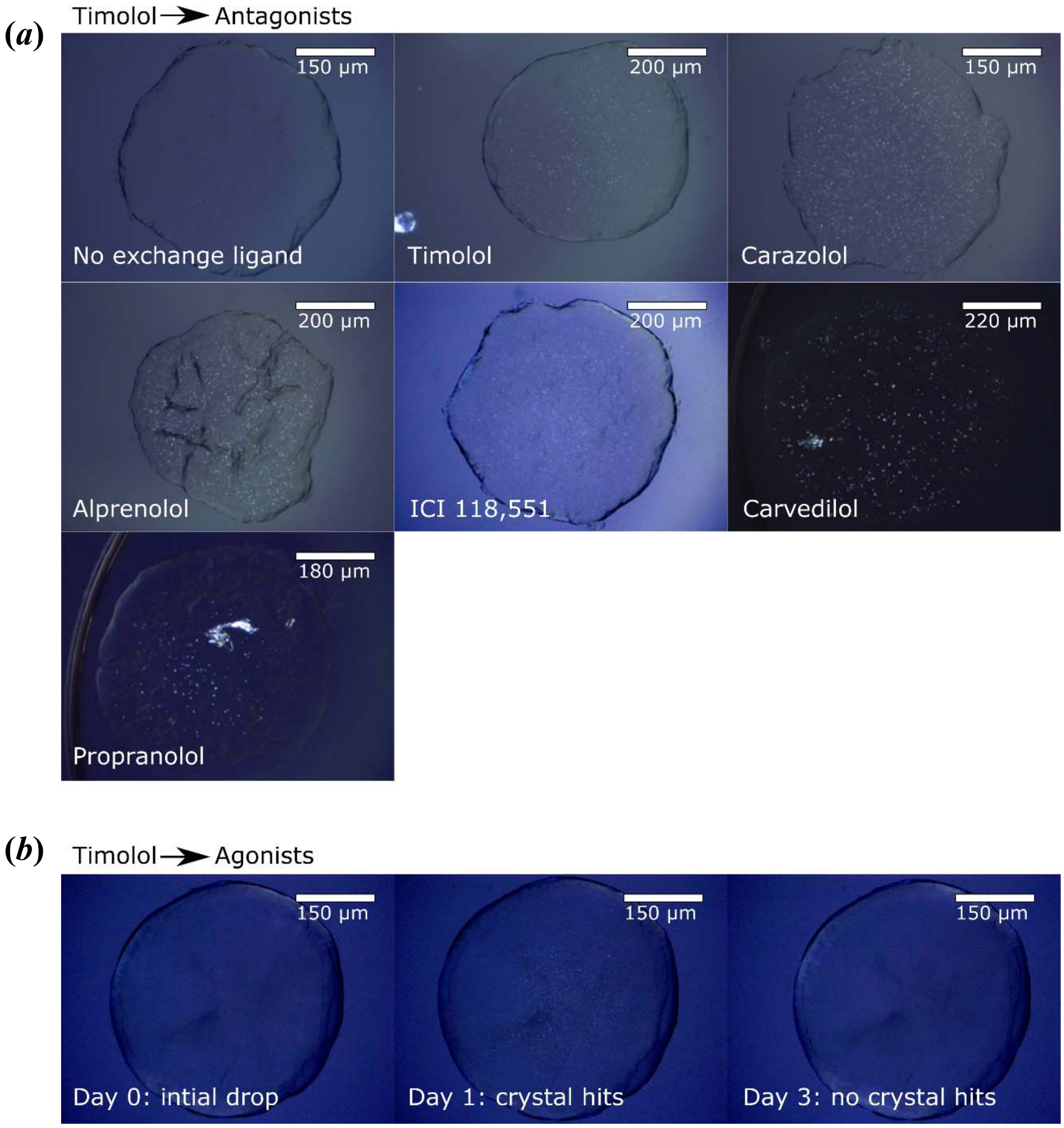

Figure S1 Ligand exchange screening experiments with β_2AR in glass sandwich plates. (*a*) Transient ligand timolol was exchanged to a variety of β_2AR antagonists. The images were taken under cross-polarized light. (*b*) Transient ligand timolol was exchanged to a β_2AR agonist formoterol. The images were taken under cross-polarized light.

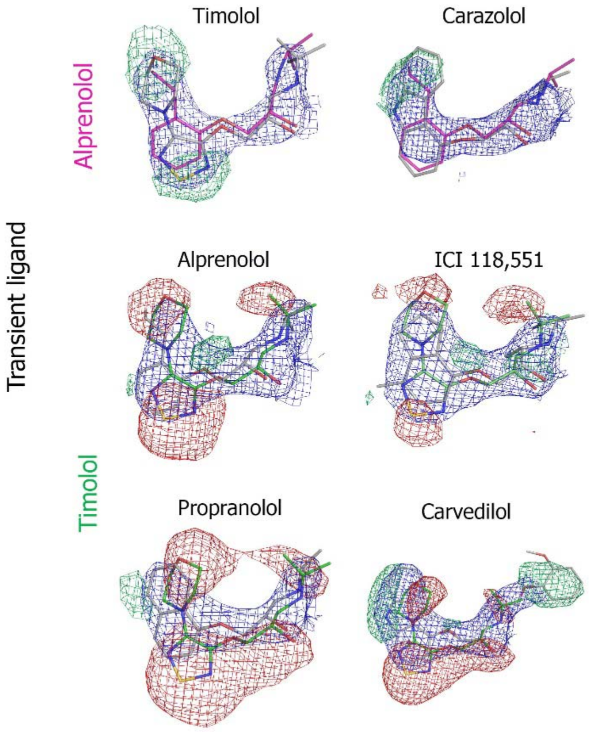

Figure S2 Cross-validation of the ligand exchange in β_2AR using crystallographic data. The maps are calculated using the corresponding transient ligand (timolol, green sticks, or alprenolol, magenta sticks) instead of the target ligands (all shown as grey sticks). Poor 2mFo-DFc electron densities (contoured at 1 σ in blue) and residual mFo-DFc electron densities (green for positive and red for negative) contoured at 3 σ clearly confirm that the transient ligand has been successfully exchanged.

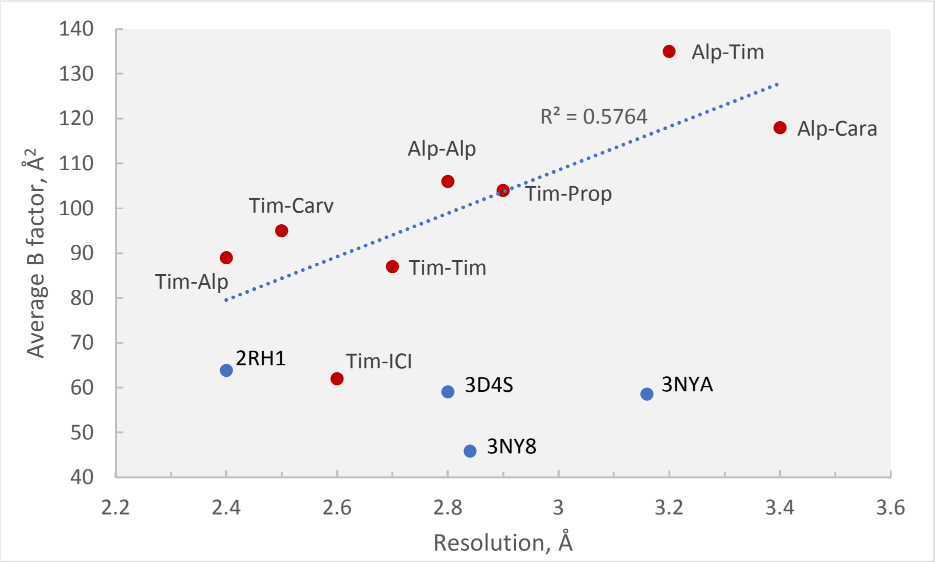

Figure S3 Correlation between the resolution and the corresponding average B-factors for the β_2AR structures obtained in this study. Red circles correspond to the structures solved at XFELs and blue circles correspond to the previously published synchrotron structures of β_2AR with various ligands (2rh1 – carazolol, 3d4s – timolol, 3ny8 – ICI118,551, 3nya - alprenolol). The linear regression fit is shown as a blue dotted line.

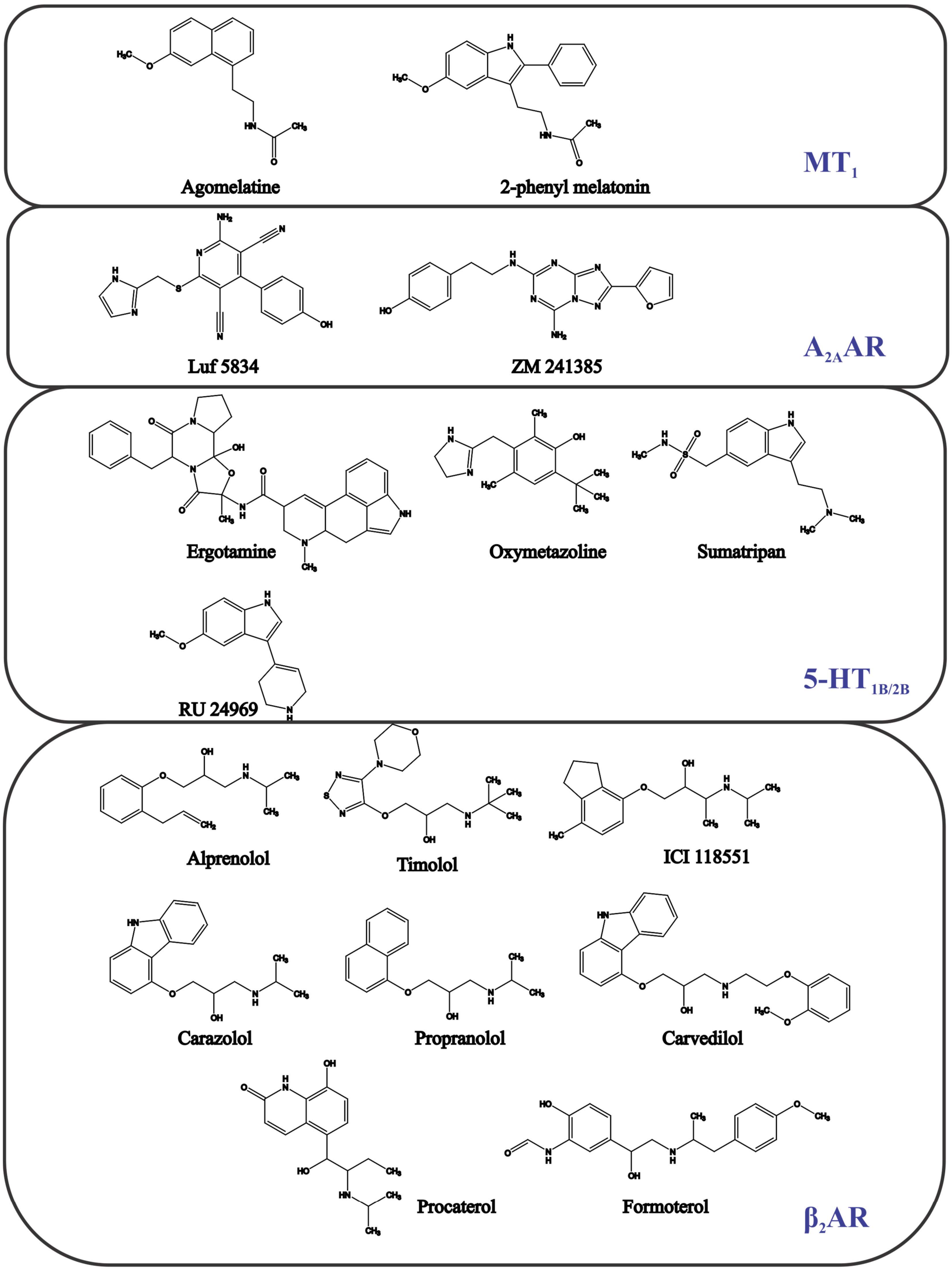

Figure S4 Chemical structures of small molecule compounds used as transient or target ligands in this study. Molecules are grouped by their specificity to a given receptor as indicated in the right bottom corner of each box.

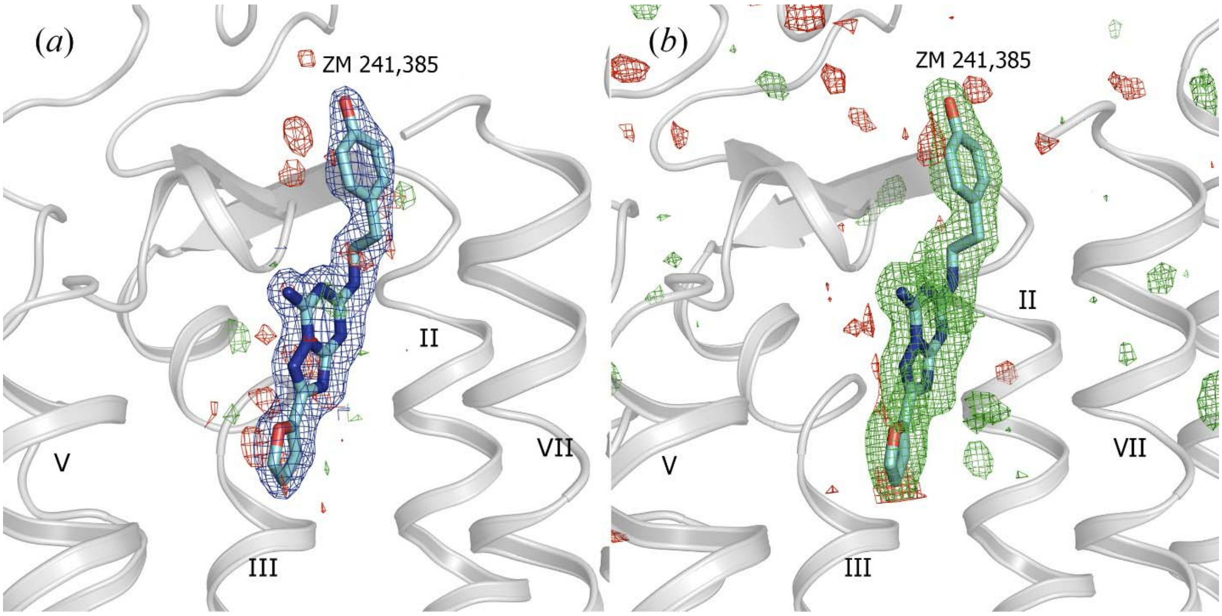

Figure S5 Ligand exchange in $A_{2A}AR$. (*a*) Electron density $2mF_o$-DF_c map (blue mesh), contoured at 1 σ, in the ligand-binding pocket of $A_{2A}AR$ after LUF5843 to ZM241385 ligand exchange. Experimental difference map $F_{o(Luf\text{-}ZM)}$-$F_{o(5K2D)}$, contoured at ±3 σ (green and red for positive and negative peaks, respectively), calculated using differences between structure factor amplitudes from $A_{2A}AR$_LUF-ZM structure and the previously solved $A_{2A}AR$_ZM241385 XFEL structure (PDB entry 5k2d), whereas the phases were taken from the $A_{2A}AR$_LUF-ZM structure. (*b*) Electron density mFo-DFc polder ligand omit maps (Liebschner *et al.*, 2017) contoured at ±3 σ in the ligand-binding pocket of $A_{2A}AR$ after LUF5843 to ZM241385 ligand exchange. Positive and negative electron density peaks are shown in green and red, respectively.
